# Determination of Leaf Water Content by Visible and Near-Infrared Spectrometry and Multivariate Calibration in *Miscanthus*

**DOI:** 10.3389/fpls.2017.00721

**Published:** 2017-05-19

**Authors:** Xiaoli Jin, Chunhai Shi, Chang Yeon Yu, Toshihiko Yamada, Erik J. Sacks

**Affiliations:** ^1^Department of Agronomy and the Key Laboratory of Crop Germplasm Resource of Zhejiang Province, Zhejiang UniversityHangzhou, China; ^2^Division of Bioresource Sciences, Kangwon National UniversityChuncheon, South Korea; ^3^Field Science Center for Northern Biosphere, Hokkaido UniversitySapporo, Japan; ^4^Department of Crop Sciences, University of Illinois, Urbana-ChampaignUrbana, IL, USA

**Keywords:** *Miscanthus*, leaf water content, drought-resistant breeding, VIS/NIR spectroscopy, sensitive wavelengths

## Abstract

Leaf water content is one of the most common physiological parameters limiting efficiency of photosynthesis and biomass productivity in plants including *Miscanthus*. Therefore, it is of great significance to determine or predict the water content quickly and non-destructively. In this study, we explored the relationship between leaf water content and diffuse reflectance spectra in *Miscanthus*. Three multivariate calibrations including partial least squares (PLS), least squares support vector machine regression (LSSVR), and radial basis function (RBF) neural network (NN) were developed for the models of leaf water content determination. The non-linear models including RBF_LSSVR and RBF_NN showed higher accuracy than the PLS and Lin_LSSVR models. Moreover, 75 sensitive wavelengths were identified to be closely associated with the leaf water content in *Miscanthus*. The RBF_LSSVR and RBF_NN models for predicting leaf water content, based on 75 characteristic wavelengths, obtained the high determination coefficients of 0.9838 and 0.9899, respectively. The results indicated the non-linear models were more accurate than the linear models using both wavelength intervals. These results demonstrated that visible and near-infrared (VIS/NIR) spectroscopy combined with RBF_LSSVR or RBF_NN is a useful, non-destructive tool for determinations of the leaf water content in *Miscanthus*, and thus very helpful for development of drought-resistant varieties in *Miscanthus*.

## Introduction

*Miscanthus* is a class of grass species, some of which have high potential of biomass productivity and could be used as a feedstock of renewable energy (Zhao et al., 2014; Yu et al., 2015). *M. sacchariflorus, M. sinensis*, and *M. fIoridulus* belonging to subtribe *Saccharinae* were proved the most potential biomass grass species (Xi and Jeźowski, 2004). These species generally grow in the similar environment; even grow together (Xi and Jeźowski, 2004; Clark et al., 2016). In China, the bioenergy *Miscanthus* crop is proposed to be planted on the marginal lands where water is deficient, so the drought-tolerant genotypes or varieties are needed for this type of lands (Dai et al., 2013; Yu et al., 2015). Water deficit is one of the major factors limiting biomass productivity in crops. Leaf water content is a very important parameter in determination of plant drought and salinity tolerance, because water stress restricts transpiration including closure of stomata and water evaporation from leaf surface. Water stress also affects crop photosynthesis and productivity (Shirley et al., 1990; Arndta et al., 2015). The genotypes with tolerance to drought and salinity in barley showed stable water content and more dry matter (Nevo and Chen, 2010; Ahmed et al., 2013). Moreover, the leaf water content was used for guiding crop fertilizer application and irrigation, even for remote sensing (Hunt and Rock, 1989). Thus, leaf water content is very important for crop management.

Today, the classical assessment of water content is based on the weight change between fresh and dried leaves. It is certainly destructive and time-consuming. Meanwhile, detection of plant water stress caused by drought is a major goal for remote sensing in the field. Determination of plant water stress by remote sensing has been proposed using indices of Near-Infrared (NIR, 0.7–1.3 μm) and Middle-Infrared (MIR, 1.3–2.5 μm) lights (Hunt and Rock, 1989). But the natural variation in relative water content (RWC) under water stress is about 20% for most plants, and thus the indices derived from NIR and MIR reflectance cannot be used to remote-sense of water stress. Recently, Near-Infrared spectroscopy analysis has been extensively studied for measurement of water content. NIR spectroscopy is frequently used for rapid and reliable prediction of quality parameters in plant, food, animal, and pharmacy (Prevolnik et al., 2011; Lin et al., 2014; Aernouts et al., 2015; Wahid et al., 2015). Lin et al. (2014) successfully developed four calibration models of grain protein content (GPC) in barley, which could be applied to quality control in malting, feed processing, and breeding selection. NIR measurement with subsequent sorting is usually based on the chemical composition of materials, but it has also been used for more physical parameters such as the gross meat content of intact crabs (Wold et al., 2010). A prediction performance for unpeeled potatoes (*R*^2^ = 0.92, RMSECV = 1.06) was obtained with the on-line measurement configuration, showing the possibility of using the instrument for the on-line measurement (Helgerud et al., 2015).

Likewise, there were several reports on water content in plant, food, animal, and pharmacy. Accurate determination of residual moisture content in a freeze-dried (FD) pharmaceutical product is critical for prediction of its quality. The multivariate modeling of moisture content in freeze-dried mannitol-containing products were constructed by NIR spectroscopy (Yip et al., 2012). The near-infrared hyperspectral imaging was applied to predict the water content of golden pothos (*Epipremnum aureum*) leaves, after which partial least square regression (PLSR) analysis was performed to predict the averaged water content (Higa et al., 2013). A good-quality model of moisture content was constructed with a root mean square error of cross validation of 0.90% (*R*^2^ = 0.99) for the straws of the *Miscanthus* × *giganteus*, a triploid hybrid of *M. sacchariflorus* and *M. sinensis*, and the short rotation coppice willow (Fagan et al., 2011). All in all, NIRS was used to successfully estimate several key quality parameters including water content, moisture, dry matter, ash, and protein content (Boschetti et al., 2013).

In order to develop relationship between spectral data and analyzed objects, several multivariate calibration algorithms were applied. Partial least squares (PLS) regression is linear algorithm, which obtain good performance when there was a linear relationship between spectra and properties of objects (Shao et al., 2010). PLS regression have been widely used in the determination of NIR and properties of objects. Recently, two non-linear regression models including least squares support vector machine regression (LSSVR) and artificial neural network (ANN) were popular. LSSVR is an interesting reformulation of the standard support vector machine (SVM) simplified by Suykens and Vanderwalle (1999). It develops models by small samples, non-linearity, and high dimension with a good generalization performance. Moreover, ANN also deals with non-linear regression, but many parameters such as hidden layer size, learning rate, and momentum have been to be set using ANN algorithm (Despagne and Massart, 1998). Above all, the near-infrared spectroscopy has the potential to predict water content. However, no model for leaf water content was explored so far in multiple *Miscanthus* species. Therefore, the aim of the current study was to investigate and evaluate application of the near-infrared instrument in determination of leaf water content in multiple *Miscanthus* species with diverse geographical origination and big sample size.

## Plant materials and methods

### Sample preparation

A total of 624 *Miscanthus* samples consisting of 167 *M. sinensis*, 169 *M. sacchariflorus*, 120 *M. lutarioriparia*, 166 *M. fIoridulus*, and 2 *M*.×*giganteus* were collected from *Miscanthus* fields in three China provinces, Zhejiang (Zhuji, E120°09.441′, N29°49.509′), Hubei (Changsha, E113°04.08.4′, N28°11.14.6′), and Hunan (Wuhan, E113°04.08.4′, N28°11.14.6′; Table S1). The detailed information regarding the samples is listed in the Table S1. Fresh leaves were taken from each sample, sealed in plastic bags and stored at 4°C under dark condition before scanning.

### Water content analysis

The fresh leaves of each sample were weighed and record as Wf, then dried at 104°C for 2 and 72 h at 80°C. The dry matter weighed was record as Wd. The leaf water content was calculated as the following:

$$\begin{array}{l} \text{Water content}\left(\%\right)=\left(\text{Wf}-\text{Wd}\right)/\text{W}{\text{f}}^{*}100 \end{array}$$

Where, Wf, fresh weight and

            Wd, dry weight.

Each sample was measured in biological triplicate.

### Measurement of near-infrared spectroscopy

The fresh leaves of about 2.5 g in weight were loaded into a circle sample cup (35 mm in diameter and 18 mm in depth) and pressed slightly to obtain similar packing density. All the samples were scanned in transmission mode (400–2,500 nm) with an interval of 2 nm using a scanning monochromator FOSS NIRSystems 6500 (FOSS NIRSystems, Silver Spring, MD, USA) in reflectance mode. Spectral data were collected using Vision software (version 3.5.0.0). Thirty-two scans were performed for each sample. In addition, each sample was loaded and scanned three times, and the average spectrum of each of the three recordings was used for NIR analysis. To avoid bias in subset partition, all samples were first arranged in an ascending order according to their respective water content values. Then one sample was picked out in order from every three genotypes. This process resulted in the prediction set of 208 samples for the validation, and the calibration set of the remaining 416 samples.

### Data processing and analysis

#### Spectral data pre-treatment

In order to improve quantity of the spectra and reduce the systematic noise, some spectral preprocessing methods were applied. The procedure for pretreatment embedded in the Unscrambler V9.5 (CAMO PROCESS AS, Oslo, Norway) was carried out. The preprocessing methods including wavelet transformation (WT), smoothing, normalization, spectroscopic transformation, multiplicative scatter correction (MSC), the first derivative of the calibration spectra calculated with three gaps of data points, baseline and standard normal variance with de-trending (SNV-D) were used in this study, respectively. The effect of every pretreatment was analyzed by naked eyes and partial least squares (PLS).

#### Multivariate data analysis

Principle component analysis (PCA) was performed as a tool to extract the main information in multivariate data in this study using the Unscrambler V9.5 (CAMO PROCESS AS, Oslo, Norway). The PLS was carried out to develop a linear model for the relationship between a set of independent spectral variables (X) and a single dependent variable (Y) by Unscrambler V9.5 (CAMO PROCESS AS, Oslo, Norway).

LSSVR and RBF_NN were carried out with the embedded LSSVM toolbox of MATALAB (Version 7.8.0.347, The MathWorks, Inc., US). LSSVR presented an interesting formulation of SVM regression by a linear set of equations to obtain the support vectors. All standard LSSVR algorithms were defined by Suykens and Vanderwalle (1999). In the optimization of the modeling parameters, two parameters, γ and the σ^2^ in the RBF kernel function, should be determined before the application of RBF_LSSVR, while only γ was optimized using Lin_LSSVR model. RBF_NN is a type of non-linear neural network, evaluated by standard error of calibration (SEC), standard error of prediction (SEP), and the correlation coefficient (r) between the predicted and measured parameters. A model with a low SEC, a low SEP, and a high r was considered as a good model (Li and He, 2006). Moreover, the residual predictive deviation (RPD), defined as the ratio between standard deviation (SD) of the samples' reference values and SEC for NIR spectroscopy calibrations, was a good index to evaluate the quality of regression models (Fearn, 2002; Arana et al., 2005). A relatively high RPD value indicates that the model is able to reliably predict the chemical composition (Arana et al., 2005). SEC and SEP were defined as follows:

$$\begin{array}{l} \text{SEP}=\sqrt{\frac{1}{I\text{p}-1}{\left(\sum_{i\;=\;1}^{I\text{p}}\hat{\text{y}}\text{i}-\text{yi}-\text{bias}\right)}^{2}}\\ \text{SEP}=\sqrt{\frac{1}{I\text{c}-1}{\left(\sum_{i\;=\;1}^{I\text{c}}\hat{\text{y}}\text{i}-\text{yi}\right)}^{2}}\\ \text{bias}=\frac{1}{I\text{p}-1}\sum_{i\;=\;1}^{I\text{p}}\left(\hat{\text{y}}\text{i}-\text{yi}\right) \end{array}$$

where,

ŷi, the predicted value of the *i*th observation

yi, the measured value of the *i*th observation

*I*p, the number of observations in the testing set

*I*c, the number of observations in the calibration set

Bias, systematic difference between the predicted and observed values.

## Results and discussion

### Water content and the character of the reflectance spectra

**Figure 1:**
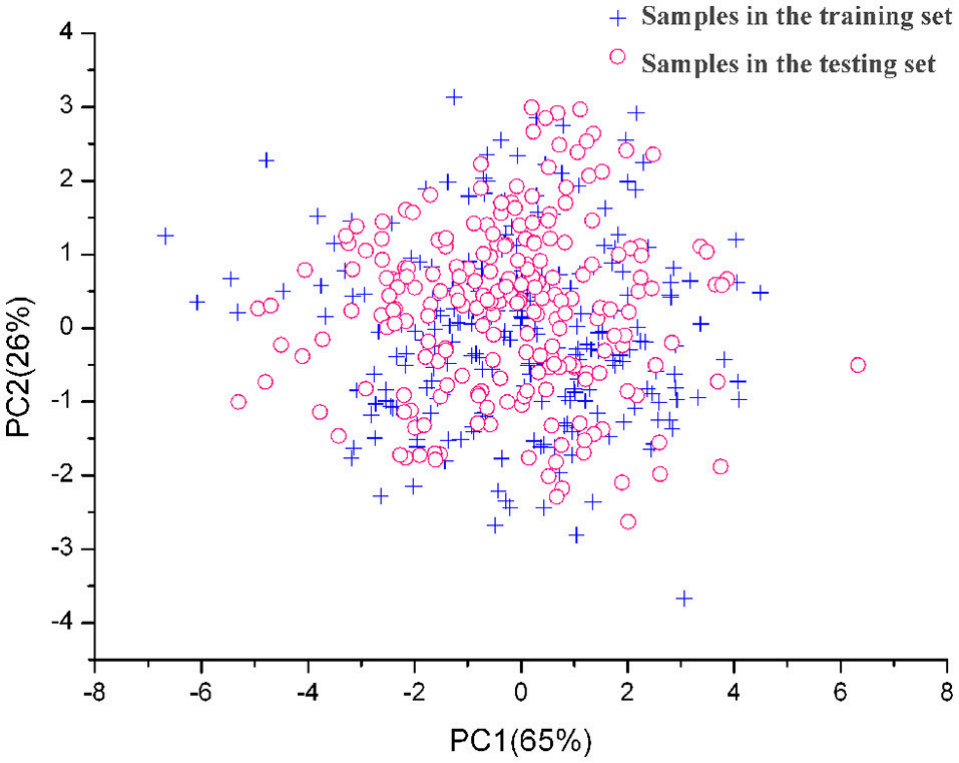
**Distribution of training samples and testing samples in principal components space**.

In our study, the 624 *Miscanthus* samples were randomly divided into two groups: a training set (416 samples) was formed to develop the calibration models, and a testing set (the remaining 208 samples) was built to validate the models (Table S1). The water content in the training/calibration set ranged from 57.77 to 82.64% with a mean of 69.55%, while the water content in the testing set varied from 58.20 to 85.94% with a mean of 74.14% (Table 1). The range of water content in the training set almost covered the testing set. Meanwhile, the testing set was evaluated using the spectral data by principal component analysis. The first and second component accounted for 65 and 26% of the raw spectral data, respectively, and could explain 91% of variation in total (Figure 1). All the samples in the testing set distributed evenly in the training set.

**Table 1:** **Statistic parameters for leaf water content in calibration and testing sets of ***Miscanthus*** samples**.

**Set**	**SN^a^**	**Minimum (%)**	**Maximum (%)**	**Mean (%)**	***SD*^b^**
Calibration set	416	57.77	82.64	69.55	4.54
Testing set	208	58.20	85.94	74.14	5.49
Total samples	624	57.77	85.94	71.08	5.33

a *Sample number*;

b *Standard deviation*.

**Figure 2:**
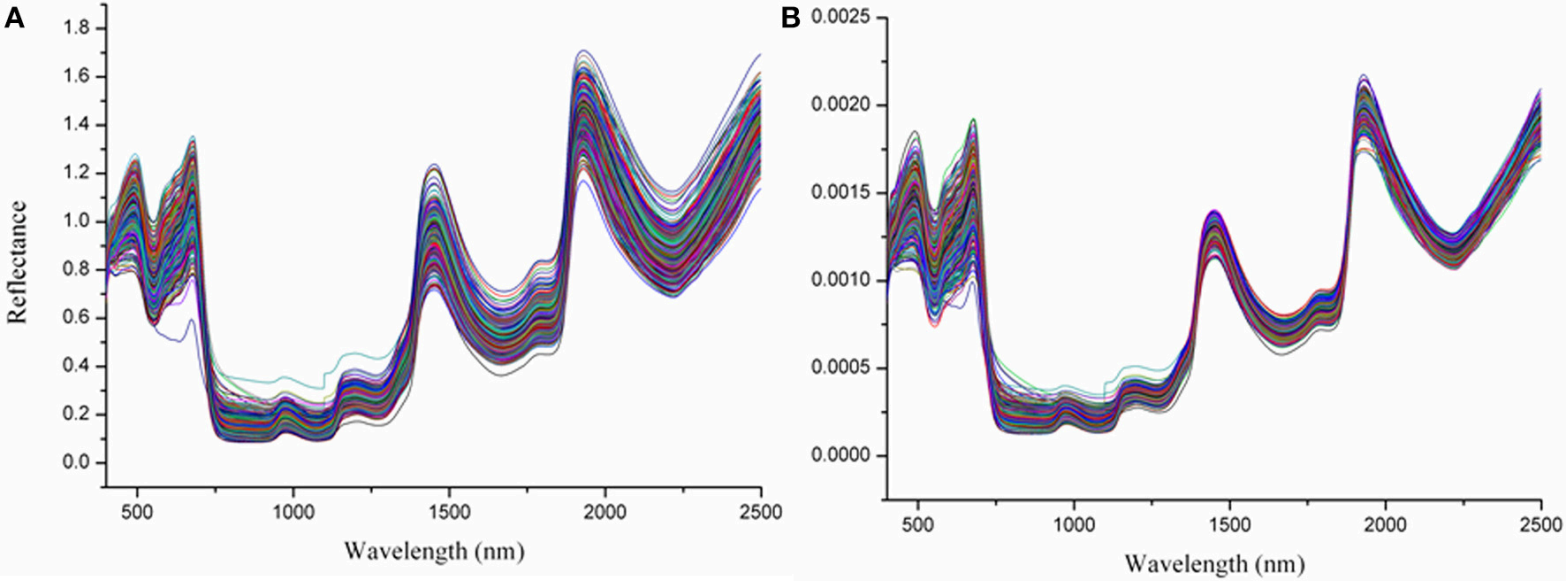
**(A,B)** Near infrared reflectance spectra of water content in *Miscanthus*, displayed by raw data **(A)**, and smoothing and normalize **(B)**.

In order to reduce background noise and improve the spectra quantity, various pre-treatment modules for determination of *Miscanthus* water content were evaluated using the Unscrambler V9.5 (CAMO PROCESS AS, Oslo, Norway; Table 2). Of all pre-treatments, smoothing, and normalization showed higher accuracy relative to the other treatments. The pre-treatments with normalization and smoothing of the spectra improved the regression performance by reducing the noise or getting a more even distribution of the variances and the average values. The normalization pre-treatment was selected for further analyses in the following models, because it had higher accuracy than the smoothing. Figure 2A exhibited the typical reflectance spectra of all samples, and the data pre-treated with normalization showed a significant change. The peaks in Figure 2B were clearer and sharper, while the NIR lines were compact. The results indicated that the data with normalization pre-treatment might be more accurate than the other pre-treatment procedures.

**Table 2:** **The evaluation of various pre-treatment models in leaf water content of ***Miscanthus*****.

**Pre-treatment**	***R*-square**	**RMSE**
Raw	0.927434	1.435722
Smoothing	0.927319	1.436861
Normalize	0.929284	1.417296
Spectroscopic	0.919114	1.515791
MSC/EMSC	0.924611	1.463382
Derivatives	0.927133	1.438693
Baseline	0.916432	0.912578
SNV	0.903898	1.652224

### Optimization for the LSSVR

Before developing the LSSVR model, the modeling parameters should be optimized.

The implementation of LSSVM requires the specification of only two parameters (γ and σ^2^). The γ is a regularization parameter, and determines the tradeoff between the structural risk and empirical risk minimization, while the σ^2^ is the kernel width parameter, playing an important role in improving the generalization performance of the LSSVR model. Furthermore, the σ^2^ is related to the confidence in the data and influences the non-linear nature of the regression. The model tends to lessen the complex solution by increasing the σ^2^, so σ^2^ reflects the distribution/range of x-values of the training data (Chauchard et al., 2004; Cherkassky and Ma, 2004). Only when the appropriate parameters are selected, the accuracy of the model can be predicted. In this study, the grid searching technique was used to optimize the two parameters. The process for optimization of the modeling parameters is to determine the minimum of a cost function with possibly multiple optima (Cherkassky and Ma, 2004). The mean of the squared residuals in the individual error (MSE) was selected as the cost function, which calculated by the standard notations as follows:

$$\begin{array}{l} \text{MSE }=\;\frac{\sum_{i=1}^{Ic}{\left(\hat{\text{y}}\text{i}-\text{yi}\right)}^{2}}{Ic} \end{array}$$

ŷi, the predicted value of the *i*th observation in the training set

yi, the measured value of the *i*th observation in the training set

*I*c, the number of observations in the training set

An optimization process of lin_LSSVR model for the water content was shown in Figure S1. The initial values of both γ and σ^2^ in the RBF_LSSVR model were set as 2 at first. The range of both γ and σ^2^ were set as 1 – 500,000. The logarithmic transformation was employed in the search plane owing to the large magnitude in the investigated ranges of these parameters. The optimal values of γ and σ^2^ were obtained with 322.4957 and 4.1720e+003, respectively, which resulted in the smallest MSE value of 0.0028.

### Accuracy comparison of LSSVR with other regression models

**Figure 3:**
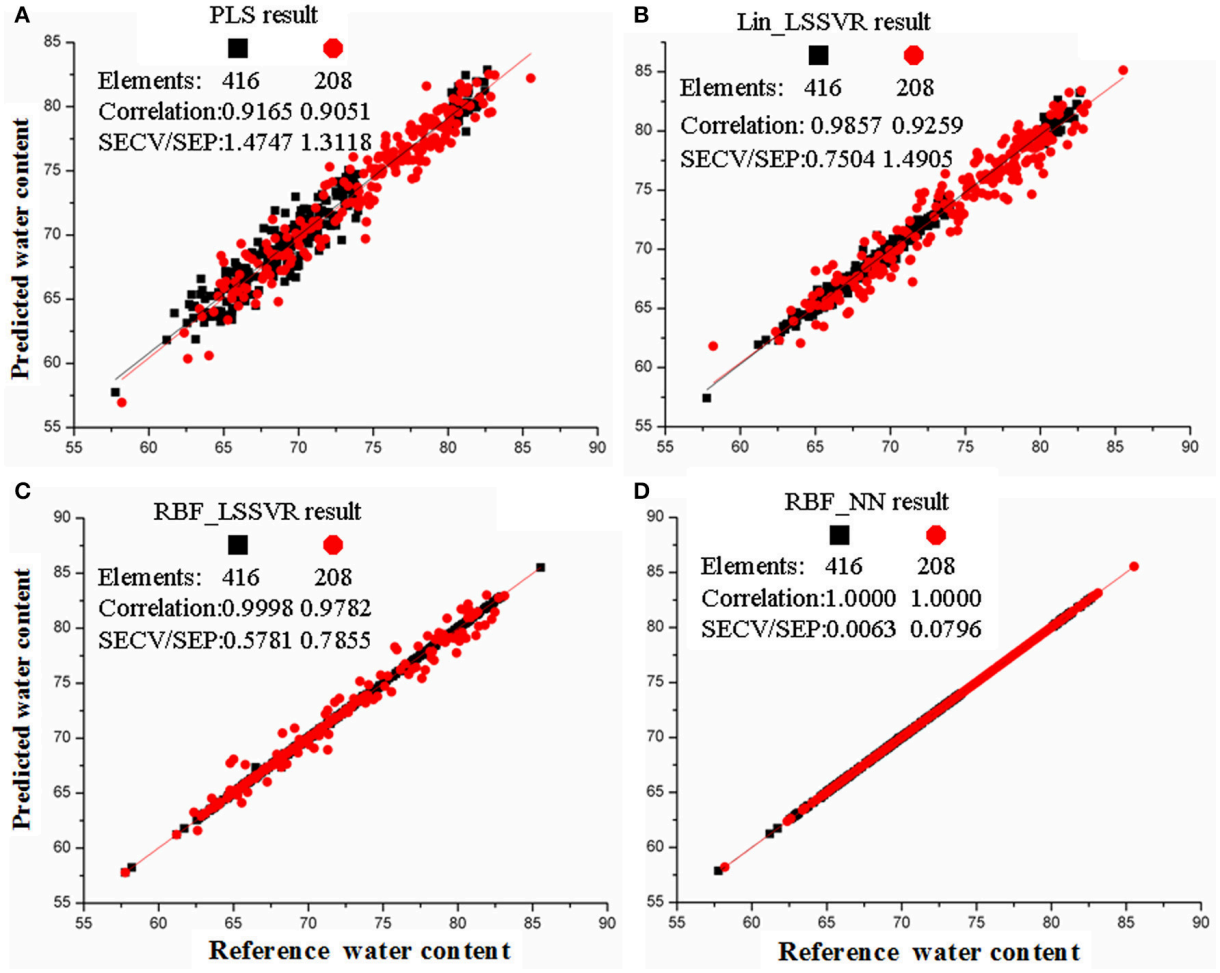
**The results of four calibration models: (A)** PLS, **(B)** Lin_LSSVR, **(C)**, RBF-LSSVR, **(D)** FBF_NN. The panes and circles represent the training samples and testing samples, respectively.

In our study, the models of PLS, lin_LSSVR, RBF_LSSVR, and RBF_NN with the same optimal parameters were developed. These four models presented good correlation between the predicted and actual water content in the correlation plots for training and testing sets (Figure 3; Table 3). In Figure 3, we found that the samples from models of Lin_LSSVR, RBF_LSSVR, and RBF_NN were more concentrated and closer to the regression lines compared with those from PLS model. Moreover, the predicted values were almost the same to the actual values in the RBF_NN model. Table 3 showed that linear determination models of PLS and Lin_LSSVR obtained lower $r_{\text{c}}^{2}$ and ${\text{r}}_{\text{p}}^{2}$, while these parameters in the non-linear determination models of RBF_LSSVR and RBF_NN were higher. The ${\text{r}}_{c}^{2}$ and ${\text{r}}_{\text{p}}^{2}$ in the RBF_NN even reached 100%. The results indicated that the non-linear determination models were better than linear determination models.

**Table 3:** **Calibration models of leaf water content corresponding to four different arithmetics using the whole and 75 sensitive wavelengths in ***Miscanthus*****.

**Wavelength**	**Model**	**Full cross-validation**		**Testing set validation**	
		**rc^2^**	**SEC**	**rp^2^**	**SEP**
400–2,500 nm	PLS	0.9051	1.4747	0.9165	1.3118
	Lin_LSSVR	0.9857	0.7504	0.9259	1.4905
	RBF_LSSVR	0.9998	0.5782	0.9782	0.7855
	RBF_NN	1.0000	0.0063	1.0000	0.0796
Sensitive wavelengths	PLS	0.9177	1.3024	0.9058	1.3969
	Lin_LSSVR	0.9579	1.0714	0.9517	1.1691
	RBF_LSSVR	0.9831	0.6823	0.97169	0.8952
	RBF_NN	0.9899	0.0136	0.9868	0.1536

*Sensitive wavelengths: 11, 14, 17, 21, 34, 64, 80, 91, 102, 108, 115, 120, 131, 140, 149, 156, 164, 175, 264, 279, 336, 348, 353, 365, 373, 382, 391, 408, 421, 439, 461, 485, 499, 511, 530, 565, 593, 627, 641, 647, 655, 661, 670, 721, 750, 759, 761, 765, 785, 806, 825, 838, 850, 855, 863, 886, 917, 927, 939, 949, 958, 965, 985, 999, 1,006, 1,008, 1,014, 1,016, 1,021, 1,023, 1,026, 1,029, 1,032, 1,039, 1,041 nm*.

### Sensitive wavelengths for determination of leaf water content in *Miscanthus*

Although, we have constructed good models in determining leaf water content in *Miscanthus*, we still need to know which wavelengths are the most sensitive for the determination. In this study, we tried to figure out the sensitive wavelengths closely related to water content in *Miscanthus*. If models are constructed using the whole wavelength data, redundant wavelengths insensitive to leaf water content would be used to calculate the predicted values. This whole wavelength-based prediction should be an cost-ineffective process requiring extensive computational time without any gain in precision. Thus, it is helpful to figure out the contributions of individual wavelengths to measurement values in *M. sinensis*. According to the theory of Haaland and Thomas (1988), the wavelengths with a large absolute regression coefficient were selected as the sensitive wavelengths. Thus, the wavelengths with sharp peaks and valleys were considered sensitive to the leaf water content in *Miscanthus* (Figure S2). In total, 75 sensitive wavelengths were found, and they yielded a significant positive correlation between the reference value and VIS/NIR spectra and had larger contribution to the calibration model for leaf water content in *Miscanthus* (Table S2).

Generally, spectral interval between 1,888 and 1,956 nm has been associated with the second overtone for biding O-H, while the second overtone and combination for free-OH located on 960–980 and 1,920–1,980 nm, respectively (Haaland and Thomas, 1988; Suykens and Vanderwalle, 1999; Fearn, 2002; Chauchard et al., 2004; Cherkassky and Ma, 2004; Tran and Grishko, 2004; Arana et al., 2005; Frost et al., 2007; Fagan et al., 2011). In the current study, 75 wavelengths were identified as sensitive and important for determination of leaf water content in *Miscanthus* (Figure S1; Table S2). The spectra with 926 and 956 nm of wavelength correspond to the 2nd overtone of free O-H, as well as 1,214 nm for the 2nd overtone of binding O-H, while the spectra at wavelengths of 1,320, 1,368, 1,396, 1,420, and 1,458 nm are associated with the 1st overtone of free O-H. Meanwhile, the wavelengths of 1,928 and 1,940 nm displayed the combination of O-H and other molecules. Furthermore, the bands around 2,203–2,237 nm of wavelengths in humite minerals are the combination of fundamental bands due to Si-OH bonding (Kronenberg, 1994). The observation of combination bands in NIR spectra of *Miscanthus* shows bands at wavelengths of 2,232, 2,252, 2,275, 2,296, 2,314, 2,328, 2,368, and 2,396 nm. The band centered at 2,440 nm was the same as that of norbergite and alleghanyite (Frost et al., 2007). In addition to these 19 wavelengths mentioned above, the other 56 wavelengths were also associated with leaf water content in *Miscanthus*, and were not observed in other materials. This result may imply the complex feature of leaf water content in *Miscathus*. In order to evaluate the actual contributions of above wavelengths, these 75 wavelengths and leaf water content were set as the independent variables and the dependent variable, respectively. The models for leaf water content were built up after 10-fold cross-validation. When the accuracy of a developed methodology is evaluated, the linear regression between reference and predicted data is usually applied (Table 3). Both the training and testing sets showed a significant correlation between the predicted leaf water content and the training result (${\text{r}}_{c}^{2}$ = 0.9177 and 0.9579 for PLS and Lin_LSSVR model, respectively; Table 3). Furthermore, the ${\text{r}}_{c}^{2}$ for non-linear models including RBF_LSSVR and RBF_NN were 0.9831 and 0.9899, while the ${\text{r}}_{\text{p}}^{2}$ of testing validation for two models were 0.97169 and 0.9868, respectively. Obviously, not only for the models of all wavelengths but also for the 75 sensitive wavelengths, the non-linear models showed higher accuracy than the linear models, indicating that the non-linear models were more suitable for determination of the leaf water content in *Miscanthus*. Even though the accuracy of the models decreased using the 75 sensitive wavelengths compared with the models based on all the wavelengths, the models based on the 75 sensitive wavelengths still presented a reasonable high accuracy. According to the analysis above, the calibration model built using the 75 sensitive wavelengths was more stable and had high prediction capability in *Miscanthus*. It provided the theory basis for the portable instrument development to detect the leaf water content rapidly and non-destructively.

In China, the bioenergy *Miscanthus* crop is proposed to be planted on the marginal lands, and thus often subjects to drought stresses (Dai et al., 2013; Yu et al., 2015). The drought-tolerant genotypes or varieties are needed for this type of marginal lands. Using the models built up with 75 sensitive wavelengths could be applied to development of the drought-tolerant *Miscanthus* varieties.

## Conclusions

We explored the feasibility of VIS/NIR spectroscopy for determination of leaf water content in *Miscanthus*. The smoothing and normalization pretreatments were the best procedures to reduce background noise and enhance quality of the spectra. The multivariate calibrations PLS, lin_LSSVR, RBF_LSSVR, and RBF_NN based on whole and the 75 sensitive wavelengths were developed for determination of leaf water content in *Miscanthus*. The RBF_LSSVR and RBF_NN models demonstrated higher accuracy than the linear models including PLS and Lin_LSSVR based on both the whole wavelengths and the 75 sensitive wavelengths. Although optimization of RBF_LSSVR and RBF_NN parameters including grid search approach and 10-fold cross-validation cost long time, it was still very effective to establish good-quality models for leaf water content in *Miscanthus*. Thus, the non-linear models based on these spectra sensitive to leaf water content could be used to develop a simple, low-cost, and effective instrument and determine the leaf water content rapidly and non-destructively in *Miscanthus*.

## Author contributions

Conceived and designed the experiments, performed the experiments, and analyzed the data: XJ. Contributed reagents/materials/analysis tools: XJ, CS, ES, CY, and TY. Wrote the paper: XJ and CS.

### Conflict of interest statement

The authors declare that the research was conducted in the absence of any commercial or financial relationships that could be construed as a potential conflict of interest.

## Supplementary material

Table S1 **Information of *Miscanthus* species, amount, and locations in the sampling regions**.

Table S2 **The regression coefficients for the 75 sensitive wavelengths**.

Figure S1 **Contour plot of MSE for grid-research and 10-fold cross-validation of RBF_LSSVR model**. The γ is a regularization parameter, and σ^2^ is the kernel width parameters. The optimal range of the parameters was determined in the first step of the grid search, and a comparatively large step width in a 10 × 10 grid represented as “^·^” was applied. The much smaller step width was used to obtain the optimal combination of these parameters, and the search grid “^×^” is also shown. n Regression coefficient plot of the linear LSSVR model.

Figure S2 **Regression coefficient plot of the linear LSSVR model**.
