# Supplementary material for: Determination of Leaf Water Content by Visible and Near-Infrared Spectrometry and Multivariate Calibration in Miscanthus
Source: Front Plant Sci. 2017 May 19;8:721. doi: 10.3389/fpls.2017.00721 (PMC5437372; doi:10.3389/fpls.2017.00721)
Supplement: Table S1 — Information of Miscanthus species, amount, and locations in the sampling regions. [file Table1.DOCX]

Table S1 Information of *Miscanthus* species, amount and locations in the sampling regions

| Growth location | Species | Origin | SN | Latitude | Longitude | Elevation (m) |
| --- | --- | --- | --- | --- | --- | --- |
| Zhejiang Province | *M. sinensis* | China, Japan, Korea | 102 | N29°49.509' | E120°09.441' | 56 |
|  | *M. sacchariflorus* | China, Japan, Korea, Russia | 90 |  |  |  |
|  | *M.Lutarioriparia* | China, Japan, Korea, Russia | 51 |  |  |  |
|  | *M. fIoridulus* | China | 26 |  |  |  |
|  | *M.×giganteus* | Hybrid | 1 |  |  |  |
| Hubei Province | *M. sinensis* | China | 30 | N30°09.138' | E114°17.160' | 34 |
|  | *M. sacchariflorus* | China | 49 |  |  |  |
|  | *M. Lutarioriparia* | China | 51 |  |  |  |
|  | *M. fIoridulus* | China | 40 |  |  |  |
| Hunan Province | *M. sinensis* | China | 30 | N28°11.146' | E113°04.084' | 47 |
|  | *M. sacchariflorus* | China | 30 |  |  |  |
|  | *M. Lutarioriparia* | China | 18 |  |  |  |
|  | *M. fIoridulus* | China | 100 |  |  |  |
|  | *M.×giganteus* | Hybrid | 1 |  |  |  |

SN: the number of samples
