# Supplementary material for: Determination of Leaf Water Content by Visible and Near-Infrared Spectrometry and Multivariate Calibration in Miscanthus
Source: Front Plant Sci. 2017 May 19;8:721. doi: 10.3389/fpls.2017.00721 (PMC5437372; doi:10.3389/fpls.2017.00721)
Supplement: Table S2 — The regression coefficients for the 75 sensitive wavelengths. [file Table2.DOCX]

Table S2 The regression coefficients for the 75 sensitive wavelengths

| Wavelength (nm) | Regression  coefficient | Wavelength (nm) | Regression  coefficient | Wavelength (nm) | Regression  coefficient |
| --- | --- | --- | --- | --- | --- |
| 420 | 455700 | 1162 | 242000 | 2048 | -349700 |
| 426 | -269200 | 1180 | -57720 | 2074 | 372600 |
| 432 | 526200 | 1214 | 281200 | 2098 | 319500 |
| 440 | 536100 | 1240 | -94880 | 2108 | 398200 |
| 466 | -473700 | 1276 | 118600 | 2124 | 443700 |
| 526 | -388900 | 1320 | -248300 | 2170 | -150900 |
| 558 | 162000 | 1368 | 564500 | 2232 | -729800 |
| 580 | -462300 | 1396 | -896100 | 2252 | 397100 |
| 602 | 225500 | 1420 | 654100 | 2276 | 1350000 |
| 614 | -175100 | 1458 | -245700 | 2296 | -1124000 |
| 628 | 128700 | 1528 | -150900 | 2314 | 699800 |
| 638 | -172500 | 1584 | 266900 | 2328 | 597900 |
| 660 | -374400 | 1652 | -269800 | 2368 | -397700 |
| 678 | 203600 | 1680 | -193100 | 2396 | -384200 |
| 696 | 481900 | 1692 | 219900 | 2410 | 372900 |
| 710 | -266900 | 1708 | -139200 | 2414 | -433300 |
| 726 | 496400 | 1720 | 519900 | 2426 | 284200 |
| 748 | -308200 | 1738 | -273800 | 2430 | -241400 |
| 926 | 256200 | 1840 | 342500 | 2440 | 173700 |
| 956 | -638100 | 1898 | -355100 | 2444 | -295400 |
| 1070 | 120000 | 1916 | -451900 | 2450 | 612700 |
| 1094 | -101700 | 1920 | 74330 | 2456 | -332300 |
| 1104 | 89580 | 1928 | -621600 | 2462 | 225600 |
| 1128 | 252400 | 1968 | 253300 | 2476 | -260200 |
| 1144 | -722000 | 2010 | -371500 | 2480 | 458600 |
