# Supplementary figures and images for: Determination of Leaf Water Content by Visible and Near-Infrared Spectrometry and Multivariate Calibration in Miscanthus
Source: Front Plant Sci. 2017 May 19;8:721. doi: 10.3389/fpls.2017.00721 (PMC5437372; doi:10.3389/fpls.2017.00721)

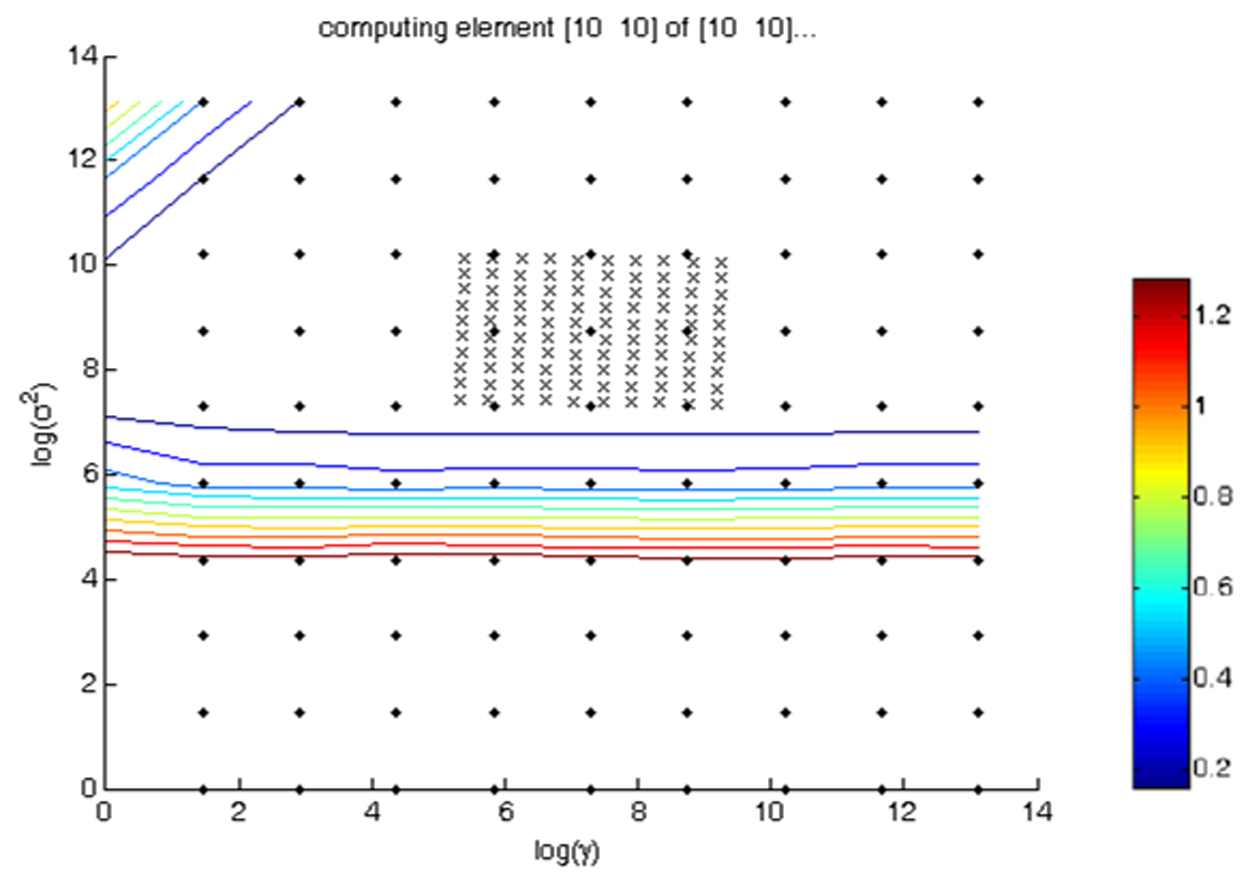

Supplement: Figure S1 — Contour plot of MSE for grid-research and 10-fold cross-validation of RBF_LSSVR model. The γ is a regularization parameter, and σ2 is the kernel width parameters. The optimal range of the parameters was determined in the first step of the grid search, and a comparatively large step width in a 10 × 10 grid represented as “·” was applied. The much smaller step width was used to obtain the optimal combination of these parameters, and the search grid “×” is also shown. n Regression coefficient plot of the linear LSSVR model. [file Image1.TIF]

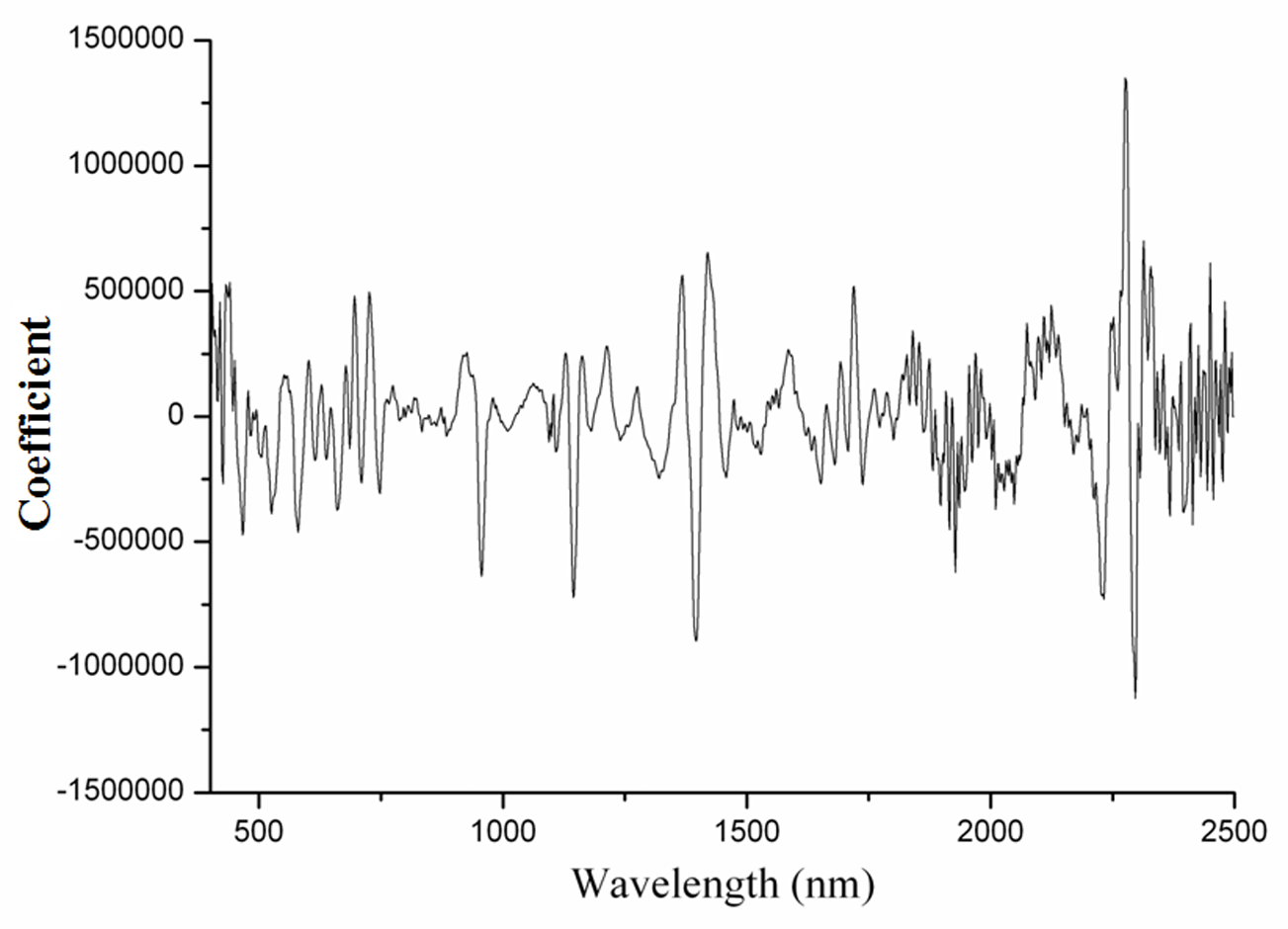

Supplement: Figure S2 — Regression coefficient plot of the linear LSSVR model. [file Image2.TIF]
